# Ally to adversary: mesenchymal stem cells and their transformation in leukaemia

**DOI:** 10.1186/s12935-019-0855-5

**Published:** 2019-05-21

**Authors:** Mugdha Sharma, Cecil Ross, Sweta Srivastava

**Affiliations:** 1 Department of Medicine, St. John’s Medical College Hospital, Bangalore, India; 2 Department of Transfusion Medicine and Immunohematology, St. John’s Medical College Hospital, Bangalore, India

**Keywords:** MSC, Leukaemia, Chronic myeloid leukaemia, HSC, Stem cell niche

## Abstract

Mesenchymal stem cells (MSC) are the key regulators of hematopoiesis. Owing to their dynamic nature; MSC differentiate into various lineages that further constitute the niche which are required for maintenance of the hematopoietic stem cells (HSC). A plethora of growth factors and cytokines secreted by MSC are essential for regulating the homeostasis within the niche in terms of cycling and quiescence of HSC. Additionally, there is a strong evidence suggesting the role of MSC in transformation of the niche to favour survival of leukemic cells. Regulation of HSC by MSC via BMP, Wnt, Notch and Sonic Hedgehog signalling has been well elaborated, however the modulation of MSC by HSC/LSC is yet unresolved. The cross talk between the HSC and MSC via paracrine or autocrine mechanisms is essential for the transformation. There are some reports implicating cell adhesion molecules, growth factors and cytokines; in modulation of MSC function and differentiation. The role of exosome mediated modulation has also been reported in the context of MSC transformation however, much needs to be done to understand this phenomenon in the present context. Similarly, the role of circulating nucleic acids, a well-studied molecular phenomenon in other tumours, requires attention in their potential role in crosstalk between MSC and HSC. This review underlines the current understanding of the physiological and pathophysiological roles of MSC and its transformation in diseased state, laying stress on developing further understanding of MSC regulation for development of the latter as therapeutic targets.

## Introduction

Leukaemia’s are myeloproliferative disorders classified and characterized by proliferation of one of the blood cell lineages. Majority of leukaemia’s have a clonal origin, attributed to leukemic stem cells (LSC). LSC occupy a hierarchical position in the series of leukemogenesis, generating progenitors with disrupted functional potential. This leads to perturbation of normal blood homeostasis. It has been well reported that LSC have originated from normal hematopoietic stem cells (HSC) upon acquisition of multiple genetic hits in due course of time [1]. Over the years with technological advancement, the role of ‘hematological stem cell niche’ has garnered consideration. The bone marrow microenvironment is a complex interphase between blood and bone which is home to not only the hematopoietic stem cells but various other cells of the non-hematopoietic origin such as osteoblasts, osteoclasts, mesenchymal cells, immune cells, endothelial cells, adipocytes, CXC12 abundant reticular (CAR) cells and more [2]. The role of niche is not only to maintain the stemness of HSC but concomitantly regulate their division and differentiation for indefinite source of blood cells. Any discrepancy in this finely tuned mechanism can lead to an imbalance. Multipotent mesenchymal stem cells (MSC) have emerged as an important regulator of tumour stroma bearing both pro- and anti-tumorigenic effects. MSC have been reported to regulate normal as well malignant hematopoiesis. The transformation of niche into a pro-malignant prototype is an area of scrutiny. The failure to eradicate LSC has been an important concern in the treatment of leukaemia’s. Since they home into the niche and are regulated by various non-hematopoietic cells including MSC, targeting their interactions and regulators can provide an advancement in treatment. To exploit MSC as a potent translational target it is important to understand its physiological properties which modulate the tumour niche. In this review, we have tried to summarize and address the role of MSC in normal vs malignant hematopoiesis and their transformation in leukaemia’s.

## Mesenchymal stem cells

It was known that transplantation of bone marrow to ectopic anatomic sites reconstituted hematopoiesis via hematopoietic and adventitial structures. Pioneering experiments by Friedenstein et al. in 1970s were the first to characterize MSC as adherent cells which have the ability to form colony forming unit in vitro and are capable of reconstitution of hematopoiesis at ectopic sites [3–5]. Although, initially isolated from bone marrow; MSC have now been isolated from various tissues such as lung, peripheral blood, adipose, brain, skin, dental, endometrium and heart [6, 7]. However, till this date unlike HSC there are no specific markers that defines MSC and they comprise a heterogenous population of cells with self-renewing multipotent potential. This attribute endows them with the ability to differentiate into several lineages including osteoblastic, chondrocytic and adipocytic lineage which further support various niche cell populations.

### Properties of Mesenchymal stem cells

The dynamic nature of MSC accounts for their role in diverse biological processes such as immunomodulation, tissue regeneration and repair, anti-fibrosis, angiogenesis and survival and maintenance of other cell populations in the niche [8]. One of the most noteworthy properties of MSC is their role in immune suppression and modulation which has been reported to aid in tumour development [8]. MSC inhibit: responses to alloreactive T lymphocytes [9], proliferation and cytotoxicity of natural killer cells [10], generation and maturation of dendritic cells and proliferation and maturation of B cells [11]. They have minimal expression of MHC class II molecules and lack the expression of costimulatory molecules CD40, CD40 ligand, CD80 and CD86 which prevents them from mounting an immune response to antigen presenting cells [12]. IFN-ɣ stimulation induces the expression of MHC II but not of costimulatory molecules [12]. MSC are also known to preserve neutrophil viability and function, induce macrophage M1/M2 phenotype transformation, promote generation and induction of CD4+CD25+ or CD8+ T regs both in vivo and in vitro [13]. M1 macrophages crosstalk with MSC via CD54 molecule in the form of ‘unconventional synapse’ leading to an increase in anti-inflammatory and immunomodulatory properties of MSC [14]. Their unique ability of immunosuppression also allows their use in the treatment of Graft vs Host Disease (GVDH) in bone marrow transplantations [15]. IFN-ɣ pre-treated MSC significantly suppress GVDH upon administration during bone marrow transplant [16]. Although the exact mechanism underlying the immunosuppressive effects of MSC is still ambiguous, most studies indicate that soluble factors like prostaglandin E2 (PGE2) [17, 18], indoleamine 2,3-dioxygenase (IDO) [19], hepatocyte growth factor (HGF) [20] and transforming growth factor (TGF)-β 1 are involved [21]. Bone marrow MSC mediate contact dependent suppression of proliferation and survival of T cells while simultaneously increasing the proportion of Tregs [22].

The plasticity of MSC to give rise to multiple lineages has implicated their role in tissue regeneration and repair. Single cell RNA sequencing of bone marrow MSC indicated simultaneous expression of markers for multiple lineages however, despite lineage priming MSC may change fate due to an environmental cue [23]. Single cell transcriptome also identified three distinct subpopulations of bone marrow MSC using FGFR2, PLAT, VCAM1, FGF2 and FGF5 [24]. It has been shown that addition of MSC to an active immune environment decreases the secretion of pro-inflammatory cytokines TNF-ɑ and IFN-γ, while concurrently increasing the production of anti-inflammatory cytokines IL-10 and IL-4 [25]. MSC paracrine signalling reduces inflammation, promotes angiogenesis and induces cell migration and proliferation by secreting various chemokines, cytokines and growth factors such as VEGF, PDGF, bFGF, EGF, FGF and TGF-β [26, 27]. These factors act as mediators for proliferation, migration and gene expression changes of epithelial/endothelial cells, fibroblasts and macrophages which further aid in the repair process [28].

One of the most intriguing roles of MSC is regulation of hematopoiesis, which they coordinate by secreting a plethora of soluble factors with diverse functions such as chemoattraction, migration, homing, induction of signalling, proliferation and maintenance. The maintenance of hematopoietic stem cells in their native state requires support from the underlying stromal cells through extracellular matrix molecules and soluble factors. A complex interplay of signalling networks between MSC and HSC is needed for maintenance of niche homeostasis. It is also important to note that the MSC regulate not just the normal hematopoiesis but also contribute immensely to the tumour growth by primarily regulating the microenvironment [2].

## MSC and regulation of normal hematopoiesis

In early 1977, Michael Dexter and colleagues showed that mesenchymal stromal cells could maintain the hematopoietic stem cells in vitro [29]. In 2000’s, various laboratories showed that co-culture of MSC with HSC promotes survival and enrichment of human CD34+ stem cells [30–32]. Evidently, the co-administration of the MSC promoted the engraftment of human CD34+ stem cells in NOD/SCID mice [33]. The exact location of MSC in the bone marrow has been a subject of investigation for many years. The various markers used for characterization of MSC isolated from mice bone marrow are listed in Table 1. Ferrer et al. reported HSC to localize in the vicinity of an MSC sub-population, expressing Nestin-GFP in mice. Further ablation of these MSC resulted in a 50% reduction in the primitive HSC. The Nestin-GFP MSC had an upregulated expression of CXCL12, Kit ligand, Angiopoietin-1 and VCAM-1 [34] and were innervated by sympathetic neurons that responded to circadian release of nor-epinephrine by sympathetic nervous system. The nor-epinephrine in turn stimulated β-adrenergic fibres to repress CXCL12 expression by MSC thus allowing HSC mobilization into the blood stream [34, 35]. Bone marrow nor-epinephrine along with melatonin, TNF and Cox-2 is involved in oscillation of mice bone marrow Hematopoietic stem progenitor cell (HSPC) proliferation and differentiation during day and night time. During daytime nor-epinephrine induces the metabolic reprogramming of HSPC priming them towards their differentiation and exit whereas bone marrow HSPC repopulation at night is mediated by increased bone marrow melatonin along with Cox-2 and alpha-smooth muscle actin (αSMA) macrophages and less endothelial cell permeability. Inhibition of melatonin or Cox-2 in bone marrow at night reversed the bone marrow phenotype. Also, partial deletion of Cox-2 high or αSMA macrophage from bone marrow lead to reduction of long-term HSC levels [36]. The perivascular MSC can also be identified by the presence of other markers such PDGFRα and CD51 [37]. Several groups in parallel characterized bone marrow MSC in mice using neuron glial antigen (NG2) and the Leptin receptor (LepR) [38, 39]. Deletion of NG2 and LepR markers independently lead to a significant reduction in HSC in vivo suggesting the role of MSC in niche maintenance. In terms of functional reconstitution, all these populations of MSC are quite identical but they show a marked difference in their site of localization in the bone marrow niche and their interaction with HSC [2]. Peri-arteriolar NG2^+^LepR^−^Nes^bright^ MSC coexist with quiescent HSC (Ki67^−^) while peri-sinusoidal NG2^−^LepR^+^Nes^dim^ MSC coexist with proliferating HSC (Ki67^+^) [38]. However, another group has shown that there is no difference in the location of non-dividing and dividing HSC in the niche. They observed both dividing Ki67^+^α-catulinGFP^+^c-kit^+^ cells and non-dividing Ki67^−^α-catulinGFP^+^c-kit^+^ cells localized within 10 µm distance of sinusoids [40].

**Table 1:** Heterogenous marker expression in mouse bone marrow MSC

Markers	Location
Nestin^+ bright^	Peri-arteriolar [34, 38]
Nestin^+ dim^	Sinusoids [34, 38]
Nestin^+^ PDGFRα^+^ CD51^+^ CD146^+^	Perivascular [37]
LepR^+^ SCF-GFP^+^ CXCL12DsRed^High^ Nestin-^dim^ Prx-1^+^ PDGFRα^+^ CD51^+^	Perivascular [39]
LepR^+^ PDGFRα^+^ Sca^−^	Sinusoids (subgroup of CAR cells) [39]
LepR^−^Nestin^bright^ NG2^+^ αSMA^+^	Periarteriolar [38]
PDGFRα^+^Sca-1^+^CD45^−^Ter119^−^CXCL12^−^	Arteriolar [82]
PDGFRα^+^ Sca-1^−^CD45^−^Ter119^−^CXCL12^+^	Sinusoids (CAR cells) [83]

The extracellular matrix (ECM) molecules play a pivotal role in maintaining stemness in the niche. CXCL12 and its receptor CXCR4 play an important role in hematopoiesis. It mediates migration, growth and differentiation of HSC [41]. Studies have shown that CXCL12 is expressed by mesenchymal stem cells, endothelial cells and osteoblasts in increasing order of their expression [42]. Following deletion of CXCL12 from MSC using Prx-1-cre transgene and LepR-cre transgene, a decline in HSC numbers were observed in mice [42, 43]. Other cell adhesion molecules that are involved in crosstalk are VCAM-1 on MSC and its ligand VLA-4 on HSC, tenascin-C and integrins [44–46].

The cell cycle fate of HSC is regulated by several key molecules which maintain a balance between proliferation and quiescence. Wnt signalling between HSC and MSC is multifaceted with its numerus receptor and ligand combinations lead to activation of either canonical or non-canonical pathways. The role of Wnt in the context of HSC cycling has been a subject of debate. Wnt released from MSC has a paracrine effect on HSC quiescence mediated by an upregulation of p21 [47]. However, Ichii et al. reported Wnt 3a expression in HSC downregulated kit ligand, angiopoietin-1, CXCL12 and VCAM-1 [48]. Cross talk between Notch and Wnt signalling stabilized beta catenin on stromal cells promoting self-renewal and maintenance of HSC in stem cell niche [49]. BMP4 deficient mice show a profound reduction in number of stem cells and their functionality [2]. BMP4 can act on HSC either directly or via downstream mediators such as sonic hedgehog (Shh). Shh induces cytokine induced proliferation of HSC [2]. MSC also produce cytokines such as IL-6, IL-11, SCF, TPO, Flt-3 ligand, CXCL12, G-CSF, GM-CSF and M-CSF to sustain hematopoiesis in vivo [50].

## MSC and regulation of leukaemia’s

### De novo modification

**Fig. 1:**
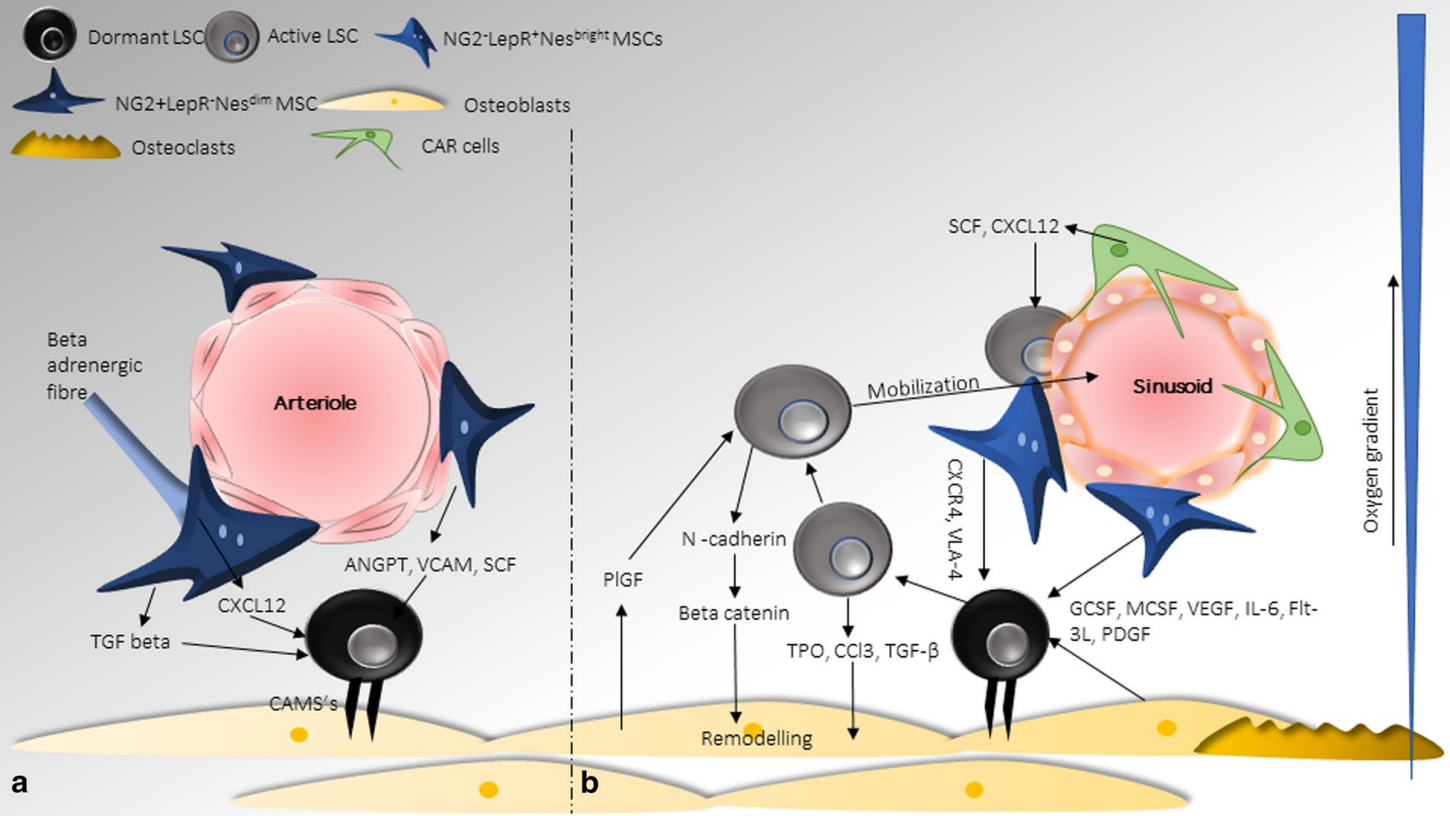
Modulation of LSC and MSC in the bone marrow niche: **a** maintenance of dormant LSC; **b** activation and mobilization of LSC

MSC can be transformed by acquiring genetic modifications which eventually manifests on the growth and maintenance of HSC. The origin and basis of these modifications are yet to be completely understood. The initial evidence on the role of genetically transformed stromal cells in the formation of myeloproliferative disease came from Rupec et al. who established that deletion of IkBɑ from the stromal cells of mice leads to MDS [51]. Loss of Retinoic acid receptor gamma (RARγ) gene from microenvironment exhibited an MPN-like phenotype wherein there was a reversion of the phenotype upon transplantation of bone marrow or spleen cells from RARγ^−^/^−^ mice to wild type mice, but not in RARγ^−^/^−^mice [52]. This exemplifies the role that a defective microenvironment may play in initiating neoplasms. In another study in mice, a link between deletion of Rb (Retinoblastoma) gene in both HSC as well as stromal cells established a myeloproliferative like disorder resulting in subsequent mobilization and loss of HSC. Interestingly, Rb deletion either in stromal cells or HSC alone did not manifest the disease, yet transplantation of Rb deleted HSC in Rb deficient mice lead to an induction of disease [53]. Similarly, Mib-1 (Mind bomb) deletion in MSC resulted in activation of Notch signalling in microenvironment leading to a myeloproliferative disorder. On transplantation into wild type mice, these cells did not show a similar phenotype which seems to be restricted only to Mib-1 null microenvironment [54]. Raaijmakers et al. suggested that alterations in progenitors of stromal cells can also result in hematological malignancies. They observed that deletion in Dicer1 along with reduced levels of sbds protein in mesenchymal osteo-progenitors (but not in mature osteoblasts) resulted in MDS and AML like phenotype [55]. Deletion of sbds gene in mesenchymal progenitor cells induced mitochondrial dysfunction, oxidative and genomic stress in HSPC and the damaged associated molecular pattern (DAMP) molecules s100a8 and s100a9, secreted by MSC mediated the effect [55]. Deletion of *Sipa1* in mouse bone marrow stromal cells leads to the development of MPN/MDS phenotype. MSC and progenitors in bone marrow microenvironment are physically and functionally altered due to an enhanced inflammatory cytokine profile (TGF-β, TNF-α) and dysregulation of Dicer1, KITL, ANGPT1, Il-7, CXCL-12 and Thpo. *Sipa1* gene is observed to be downregulated in leukaemia’s such as CML, CNL and CMML [56]. MSC from CML patients also cause immune suppression by reduced T-lymphocyte proliferation via granulocyte myeloid derived suppressor cell (G-MDSC). Up regulated immunomodulatory factors secreted by CML-MSC as compared to normal MSC include Cox2, TGF-β, Arg1 and IL-6, which modulate the function of the HSC [57]. Genetic, functional and immunologic characterization of bone marrow MSC from AML patients vs healthy donor bone marrow MSC showed an increase in anti-inflammatory and immunosuppressive mechanisms with increased IL-10 linked to disease outcome [58]. An altered microenvironment, due to aberrant signalling or production of pro-inflammatory cytokines can aid in the initiation and maintenance of hematological malignancies and favour the growth of leukemic cells (Fig. 1).

### Altered homing

An upregulation in adhesion or homing molecules on both LSC and MSC can lead to prolonged survival and maintenance of these cells in vivo (Fig. 1). CXCL12 and CXCR4 serve as prognostic markers in various leukaemia’s with the expression of CXCR4 being highest in promyelocytic leukaemia, myelomonocytic AML and B-lineage ALL [59]. An upregulation of VCAM-1 on MSC and its ligand VLA-4 on leukemic cells had also been reported to be involved in the maintenance of residual disease in AML and resistance to therapy via activation of NF-κB [60–62]. Knockdown of ITGA4 gene in CML murine cells led to decreased adhesion to VCAM-1, loss of CFU forming ability and cell survival [63]. Inhibition of ITGA4 using natalizumab sensitized primary ALL cells to therapy [64]. Zhang et al. observed activation of Wnt signalling pathway in CD34+ leukemic cells by N-cadherin from bone marrow MSC in CML. Human CML stem or progenitor cells co-cultured with bone marrow derived MSC showed an increased association of N-cadherin and cytoplasmic catenin. The concentration was found to be highest in imatinib treated cells thus speculating their role in chemoresistance in CML [65].

### LSC mediated transformation

There is a bidirectional signalling in niche wherein LSC modulate the stromal cells and vice versa; ultimately transforming the niche into a self-reinforcing acclamatory leukemic niche (Fig. 1). LT-HSC (long term HSC) isolated from transgenic BCR-ABL^+^ mice and CML patients exhibited decreased bone marrow CXCL12 which debilitated their homing and engraftment in CML bone marrow. G-CSF secreted by leukemic cells down-regulated the levels of CXCL12 in CML bone marrow. This led to an anomalous proliferation and differentiation of LT-HSC subsequently conferring a selective advantage for their survival in the bone marrow [66]. High placental growth factor (PlGF) level in bone marrow plasma and peripheral blood correlated with BCR-ABL load in both CML mice and patients. CML cells induce bone marrow stromal cells to overproduce PlGF, which in return stimulates proliferation, migration, metabolism of CML cells along with bone marrow angiogenesis thus setting up a favourable vicious cycle. Since PlGF acts independent of BCR-ABL pathway it’s targeting overcame imatinib resistance and prolonged survival in imatinib resistant mice [67]. In transgenic mice leukemic cells remodelled the endosteal niche into a self-reinforcing stem cell niche. The leukemic myeloid cells were shown to interact with MSC and elevated expression of thrombopoietin (TPO) and CCL3 by these cells contributed to MSC expansion which in turn modulated osteoblasts in endosteal niche. Upregulated TGF-β and inflammatory signalling with elevated levels of CXCL12, SCF, Angiopoietin and Slit2 led to a complete transformation of the endosteal niche [68]. BMP1, PDPN, Nanog, MITF, FOXO3 and MET were upregulated in CML MSC as compared to healthy controls. Most of these except for FOXO3 were upregulated in MSC isolated from molecular response patients, suggesting their role in remodelling [69].

### Molecular regulators of transformation

Communication between the LSC and bone marrow microenvironment is a hallmark of malignant transformation which is either guided by cell–cell contact or by the extracellular secretory molecules which could include circulating nucleic acids, protein, metabolites or lipids. There are several mechanisms which have been explored to understand how the LSC may regulate the MSC or vice versa (Fig. 1). The role of important signalling pathways, secretory factors, exosomes and miRNA have been explored by several groups to understand this relationship.

Dysregulated signalling is a premonition of cancer progression. Several critical pathways such as Notch, Wnt, Shh, BMP and TGF-β are involved in the crosstalk between HSC/LSC and MSC. Notch along with its ligand Jagged and Delta and regulator Hes1 guides differentiation, proliferation and cell fate decisions [70]. Human AML related MSC express higher levels of Notch1, Hes1 and Jagged1 as compared to normal MSC leading to increased Notch signalling in AML cells and conferring chemoresistance [71]. Abrogation of signalling by blocking Notch receptors along with chemotherapeutic agents lowered the supportive effect of MSC towards AML cells [71].

Bone marrow MSC have been observed to deliver functional mitochondria to AML leukaemia initiating CD34+ cells in co-culture system providing an edge towards chemotherapeutic resistance and survival [72].

The other important contributors are exosomes which are released by cells of both hematopoietic and non-hematopoietic origin. They accomplish bi-directional transfer of mRNA, miRNA and several proteins between cells. The role of exosomes in the context of hematological malignancies is well explored in last few years. AML LSC-derived exosomes were enriched with Flt3-ITD, NPM1, CXCR4, MMP-9 and IGF-1R mRNA within the microenvironment [73]. In CML, exosomes stimulate stromal cells to produce IL-8 which control many downstream signalling pathways under CXCR1 and CXCR2 receptor [74]. CLL derived exosomes delivered contents to endothelial cells and MSC which induced CXCL16 and CCL2 expression [75]. Patient derived CML exosomes activate epidermal growth factor receptor (EGFR) signalling in stromal cells and further increases the expression of SNAI and its targets, MMP9 and IL8 [74]. AML derived exosomes induce expression of Dkk1 while downregulating CXCL12, KITL and IGF1 in bone marrow stromal cells ablating normal hematopoiesis [76]. Microvesicles derived from leukemic cell lines have been reported to carry the fusion transcript of the parental cell line; which have been uptaken by normal MSC upon co-culture increasing their proliferation in vitro [77]. Extracellular vesicles released by murine MSC lead to loss of HSC quiescence and expansion of murine myeloid progenitors via Toll like receptor (TLR4) activation of MyD88 adaptor protein [78].

Interestingly MSC are possessed with both immunomodulating and immunosuppressive properties. The induction of either one of these depends on a complex balance of cues collected from many stimuli including the TLR. TLRs represent an arm of the innate immunity which is involved in recognizing Pattern and Damage Associated Molecular Patterns (PAMPs and DAMPs); thereby regulating processes of inflammation, anti-apoptosis and wound healing [79]. MSC express TLR1,2,3,4,5,6,7 and 9. TLRs triggered by stimuli can affect biological processes of MSC [79]. TLR3 and TLR9 deficiency were shown to result in acute lymphoblastic T cell leukaemia (post induction with endogenous retrovirus) in mice [80, 81].

## Conclusion

There is gaining evidence highlighting the importance of niche metamorphosis in progression of leukaemia’s. Regardless of therapy, the LSC population persists and aids in disease relapse. While the intrinsic properties of LSC such as drug efflux pumps and altered signalling contribute to leukemogenesis, it is the malformed niche which ablates normal hematopoiesis and supports *leukemopoiesis* in later stages of the disease. With the advent of new technology attempts can be made to understand the signature of malignant MSC at single cell level. There is a dynamic crosstalk of cues between MSC and LSC which lead to changes in altered homing and the phenotypic expression of both cell types. Conserved intricate pathways with both suppressive and pro-oncogenic roles such as Wnt, TGF-β are reported to be dysregulated and involved in the transformation either directly or via extracellular mediators. Yet, a few unresolved questions still exist. Who initiates the transformation? Is there an epigenetic modulation of MSC which in turn transitions the niche, favouring the growth of LSC? How do these massive physiological changes circumvent the robust immune surveillance? Do MSC provide any metabolic edge to LSC over HSC in due course of disease? And most importantly, what are the main effectors mediating the crosstalk between MSC and LSC? The role of exosomes in leukaemia has been studied; however, the origin and regulation of exosomes in the background of MSC and LSC interaction needs further exploration. It would also be interesting to ascertain the significance of cell free nucleic acid (cfNA) in transformation of MSC, given their ability to transfect target cells. cfNA including miRNA or circulating DNA from LSC might integrate into MSC and influence both their functional as well as phenotypic properties. The presence of cfNA has further been established to have a prognostic significance in certain malignancies. These minute molecules might affect many developmental and regulatory processes, thereby causing a havoc in microenvironmental signalling. This would result in a paradigm shift towards progression of leukaemia. In order to target MSC the challenges that need to be addressed are appropriate characterization of human MSC and development of potent xenotransplantation models. It is of utmost relevance to unravel this lacuna and understand the altered MSC physiology in leukaemia to beget better therapeutics and management of disease.

## Data Availability

Not applicable.

## Abbreviations

Abl: Abelson
ALCAM: activated leukocyte cell adhesion molecule
ALL: acute lymphocytic leukaemia
AML: acute myeloid leukaemia
BCR: breakpoint cluster
bFGF: basic fibroblast growth factor
BMP: bone morphogenetic protein
CCL5: CC motif chemokine ligand 5
CD: cluster of differentiation
CLL: chronic lymphocytic leukaemia
CML: chronic myeloid leukaemia
CMML: chronic myelomonocytic leukaemia
CNL: chronic neutrophilic leukaemia
CSF: granulocyte macrophage colony stimulating factor
CXCL12: CXC chemokine ligand 12
CXCR4: CXC chemokine receptor 4
EGF: epidermal growth factor
FGF: fibroblast growth factor
FGFR: fibroblast growth factor receptor
Flt-3-ligand: Fms-related tyrosine kinase 3 ligand
FOXO3: forkhead box O3
G-CSF: granulocyte colony stimulating factor
GFP: green fluorescent protein
ICAM: intracellular cell adhesion molecule
IGFB2: insulin like growth factor binding protein 2
IL: interleukin
ITGA: integrin alpha 4
IFNγ: interferon gamma
LFA-3: lymphocyte function associated with antigen 3
LIF: leukaemia inhibitory factor
MCP-1: monocyte chemoattractant protein-1
M-CSF: macrophage colony stimulating factor
MDS: myelodysplastic syndrome
MHC: major histocompatibility complex
MITF: microphthalmia-associated transcription factor
MMP: matrix metalloprotease
MPN: myeloproliferative neoplasms
NFκB: nuclear factor kappa B
NPM1: nucleophosmin
PDGFRɑ: platelet derived growth factor receptor alpha
PDGF: platelet derived growth factor
PDPN: podoplanin
PLAT: polycystin-1 lipoxygenase alpha toxin
SCF: stem cell factor
SBDS: Shwachman–Bodian–Diamond syndrome
TNFα: tumour necrosis factor alpha
VCAM-1: vascular cell adhesion molecule 1
VEGF: vascular endothelial growth factor
VLA-4: very late antigen-4
